# Methotrexate Toxicity: A Simple Solution to a Complex Problem

**DOI:** 10.7759/cureus.14364

**Published:** 2021-04-08

**Authors:** Muhammad Asghar, Hasan Shoaib, Woosun Kang, Irfa Tariq, Tulika Chatterjee

**Affiliations:** 1 Internal Medicine, University of Illinois College of Medicine Peoria, Peoria, USA; 2 Internal Medicine, Sir Ganga Ram Hospital, Lahore, PAK

**Keywords:** patient-centered care, methotrexate toxicity, neutropenia, drug rash, cost-effective

## Abstract

Methotrexate is a highly effective medication that is the mainstay of treatment for numerous complex dermatological and rheumatological disorders. However, its use requires close monitoring as it has serious side effects that could be fatal if not recognized promptly. Herein, we present an interesting case of methotrexate toxicity leading to a prolonged hospital stay with resultant increase in health care cost and patient dissatisfaction. It remains of pivotal importance for primary care physicians and hospitalists to be aware of its side effect profile. As such, early recognition of methotrexate toxicity can result in earlier initiation of goal-directed therapies, leading to improved outcomes and shorter hospital stay. Patient education and effective communication between health care providers and the patient are of utmost importance in ensuring patient safety.

## Introduction

The origin of methotrexate (MTX) can be traced as far back as the year 1947. MTX is closely related to the drug aminopterin, which was used at the time to treat young children with acute leukemia. The patient’s clinical condition improved dramatically; however, just 10 days after the therapy, the patient developed severe stomatitis [1]. MTX is considered the mainstay treatment in many dermatological and systemic disorders and is the first line in the treatment for psoriasis [2]. Although it is clinically effective, MTX has common detrimental side effects such as mucositis and bone marrow suppression; therefore, patient education regarding adequate dosing is essential, and dosing requires close monitoring. Herein, we present a case of a patient who developed severe hematological side effects and dermatological findings after he inadvertently took a higher dose of MTX than prescribed.

## Case presentation

A 54-year-old Caucasian male with a past medical history of chronic kidney disease stage III, diastolic heart failure, chronic obstructive pulmonary disease, and plaque psoriasis was admitted with worsening of his psoriatic skin lesions on the extensor surfaces of the elbow and knees along with periumbilical abdominal lesions and a new extensive rash involving the groin along-with mucositis, stomatitis, and balanitis. The patient's vitals were stable on presentation with no fever. The patient has an extensive history of plaque psoriasis and was previously treated with MTX, which was almost resolved. Due to the patient’s psoriasis flaring up, he was started back on MTX at 10 mg per week, but he inadvertently took 10 mg of MTX daily for a total of six days.

**Figure 1:**
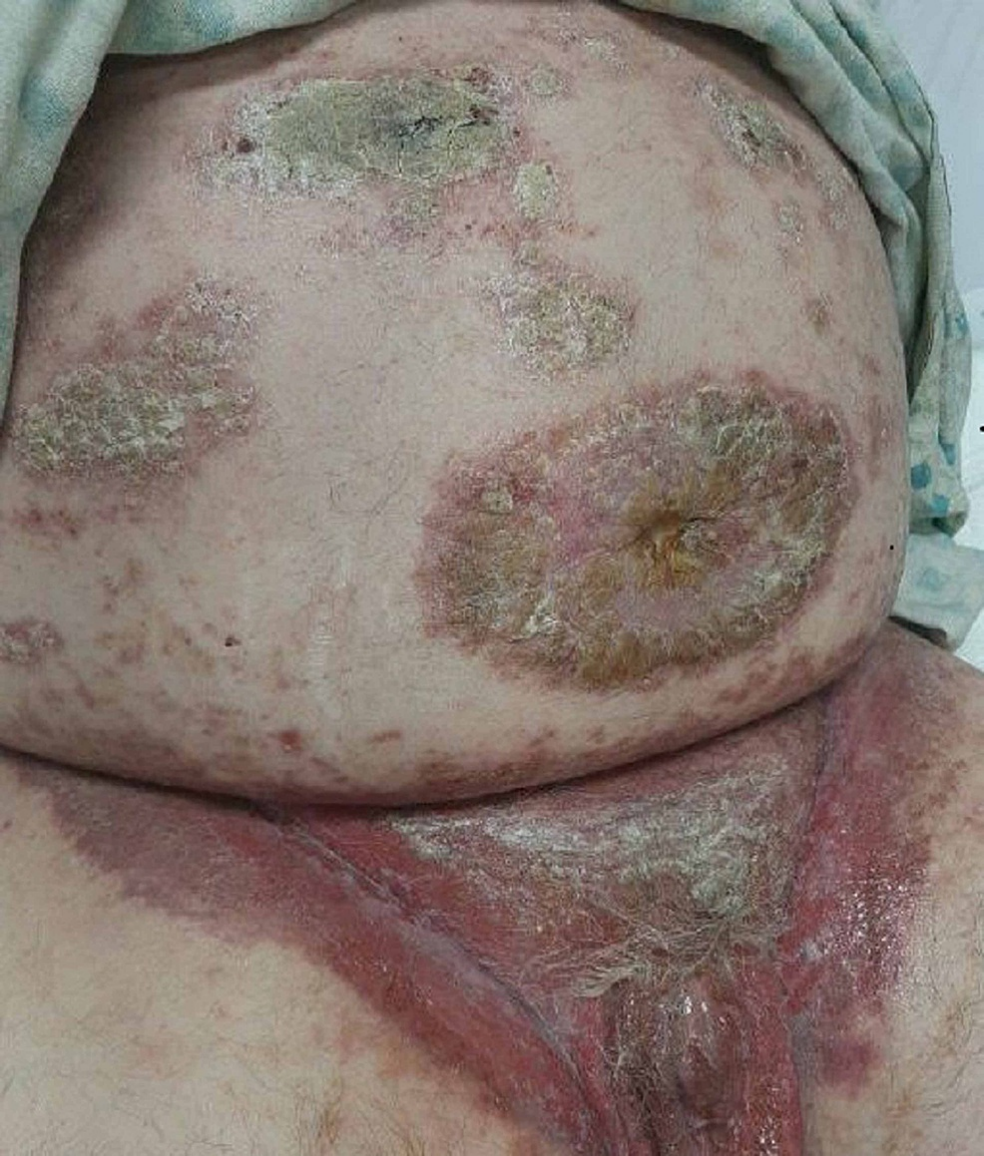
Rash involving the periumbilical and groin regions

On examination, the patient had swollen, erythematous hands and feet, making activity difficult due to severe pain. The patient also had clearly demarcated, erythematous plaque with silvery scales and pustules involving the periumbilical region, knees, and elbows. Oral examination revealed demarcated painful ulcerations in the oral cavity. The groin region had extensive erosions with desquamation of the involved skin over the erythematous base along with erosive lesions on the glans penis (Figure 1).

Given the extensive nature of his skin lesions, broad-spectrum antibiotic and antifungal coverage was started with piperacillin-tazobactam, vancomycin, and fluconazole. Biopsy of the skin lesions in the groin was performed, which showed epidermal desquamation and ulceration along with dermal infiltrate of eosinophils, which could be seen with a cutaneous rash that would arise secondary to treatment with a cytotoxic drug. Biopsy confirmed our clinical suspicion of MTX toxicity. Cultures from the periumbilical skin lesions were positive for *Staphylococcus aureus* and diphtheroid due to superimposed infection. Antibacterial were switched to cefepime while fluconazole was continued for fungal infection coverage.

The patient also developed signs of hematological toxicity associated with MTX overdose. His white blood cell count dropped down to the nadir of 0.69 x 10^3^/microliter with absolute neutrophil count (ANC) of 0.60 x 10^3^/microliter, hemoglobin of 10.1 g/dL, and platelet count of 47 x 10^3^/microliter (Table 1). The bone marrow suppression was treated with granulocyte colony-stimulating injections in our case.

**Table 1:** Showing trends of WBC, RBC, platelet, absolute neutrophil count, and methotrexate levels WBC, white blood cell; RBC, red blood cell

	6/15/2020	6/16/2020	6/17/2020	6/18/2020	6/19/2020	6/20/2020
WBC, x10^3^/microliter	1.10	1.67	2.70	2.33	1.29	1.01
RBC, x10^3^/microliter	3.49	3.68	3.89	3.77	3.61	3.75
Hemoglobin, g/dL	10.0	10.6	11.2	10.8	10.1	10.1
Platelet, x10^3^/microliter	161	112	109	81	60	47
Absolute neutrophil count, x10^3^/microliter	0.74	0.82	1.65	1.33	0.29	0.06
Methotrexate level, umol/L	0.04	0.03	0.03	0.03		

On admission, MTX was stopped and high-dose leucovorin rescue with 50 mg IV every six hours for two doses followed by 20 mg IV every six hours was initiated. NaHCO_3_ infusion of 3 liters daily was started for alkalization of urine for better excretion of MTX. MTX blood levels were measured and the patients had a level of 0.04 umol/L; on repeat testing, the MTX level was 0.03 umol/L and remained stable at 0.03 umol/L. A prednisone taper was stated at 20 mg/day for a week, tapering it to half its original dose every week until discontinuation. Given the severity of his skin lesion, cyclosporine 100 mg twice daily was added to his regimen. Adequate pain management was carried out by tylenol along with morphine.

**Figure 2:**
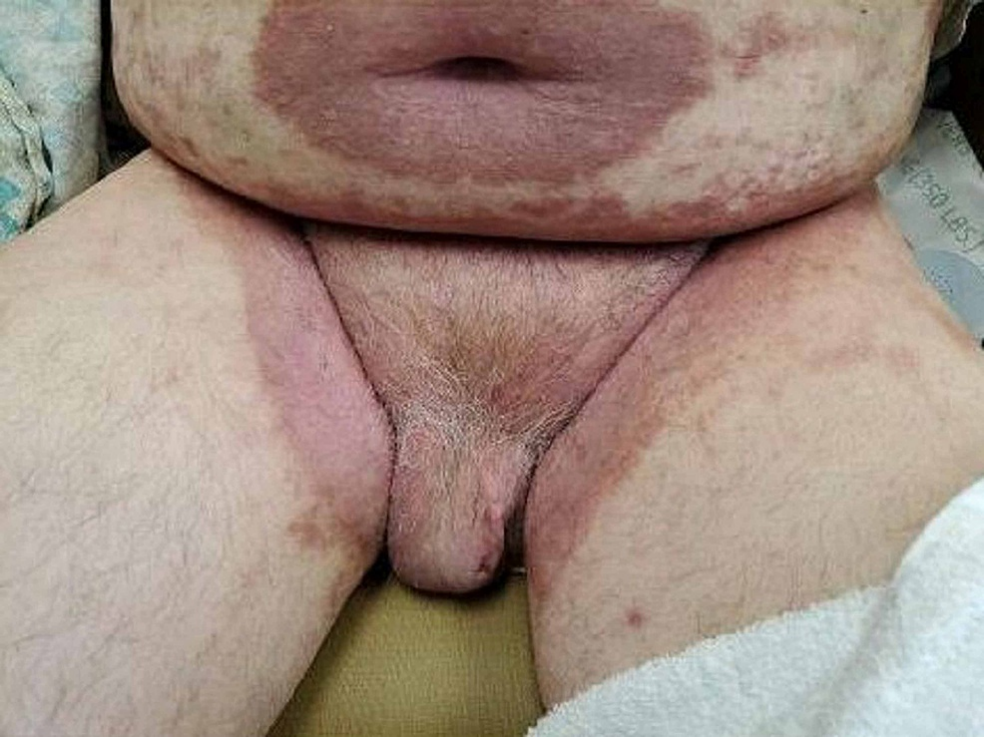
Improvement in the rash after treatment

The patient had improvement in the skin lesions with the resolution of plaques and normalization of the blood counts (Figure 2).

## Discussion

MTX, a folate analog, is absorbed in the small intestine and mainly excreted renally [3]. MTX inhibits dihydrofolate reductase due to an accumulation of polyglutamate, which prevents the synthesis of purines, pyrimidines, polyamines, and transmethylation of other compounds. Purines and pyrimidines are precursors of DNA and RNA, respectively. At high doses, MTX prevents the proliferation of cells by preventing DNA and RNA production, making it an effective tool against malignancies. The cessation of purine synthesis causes the arrest of the cell cycle in the S phase [4,5].

The variety and severity of the adverse effects is a conundrum for health care professionals in an otherwise miracle drug. While the different mechanisms of action bring out the benefits of the drug, those same mechanisms also bring out sinister effects of MTX. There is a wide spectrum of adverse effects that can be encountered; while some are reversible and depend on the dose such as stomatitis, others such as pneumonitis and bone marrow suppression are much severe [6]. Our patient presented with stomatitis and balanitis. There is a difference in acute and chronic toxicity of the drug, the latter tends to more severe than the former. Chronic toxicity is mostly considered accidental, which was reported by a study comparing the acute and chronic toxicity of the drug. Chronic toxicity is more likely to be exhibited by older individuals and by patients with renal failure, decreased albumin levels, and polypharmacy [7]. The most common cause of toxicity was due to accidental overdose or physician/pharmacist error. The majority in the chronic group experienced mucosal ulcers followed by skin lesions and gastrointestinal symptoms such as nausea, vomiting, abdominal pain, and gastrointestinal bleed. It makes sense that the group with chronic toxicity also experienced hematological abnormalities, namely thrombocytopenia, anemia, and leukopenia. This group also was a victim of hepatotoxicity due to polyglutamate accumulation [7]. The hematological picture of our patient was quite similar since the lab work showed thrombocytopenia, neutropenia, and leukopenia. The decreased cell counts across all cell lineages was also the most common adverse effect of MTX toxicity among a cohort of 28 patients in the study by Kivity et al. Low-dose toxicity, like our case, can be fatal due to bone marrow suppression [8].

Treating the complexities of the adverse effects of MTX is quite a challenge. Different treatment modalities include administering leucovorin, granulocyte colony-stimulating factor, sodium bicarbonate, and blood products depending on the signs and symptoms of the patient. The guidelines for treating an oral overdose of MTX include gastric lavage, activated charcoal, leucovorin, and sodium bicarbonate [7]. Our treatment plan was quite similar for the patient. The drug levels were monitored, but a cohort study by Kivity et al. did not show any correlation between the drug levels and decreased cell counts [8].

## Conclusions

Our case shows how a commonly used effective medication can turn into poison if not used properly. The vast side effect profile is well documented in the literature, yet we continue to deal with such cases of MTX toxicity. Most of the cases are due to accidental overdose, where the fault can lie on either side of the aisle. It is exhausting knowing the fact that these cases are preventable, thereby being burdensome to health care and on the patient. The way forward lies in adequate patient education and improvement in communication channels between different health care teams.

## References

[1] Farber S, Diamond LK (1948). Temporary remissions in acute leukemia in children produced by folic acid antagonist, 4-aminopteroyl-glutamic acid. N Engl J Med.

[2] Menter A, Korman NJ, Elmets CA (2009). Guidelines of care for the management of psoriasis and psoriatic arthritis: section 4. Guidelines of care for the management and treatment of psoriasis with traditional systemic agents. J Am Acad Dermatol.

[3] Desmoulin SK, Hou Z, Gangjee A, Matherly LH (2012). The human proton-coupled folate transporter: biology and therapeutic applications to cancer. Cancer Biol Ther.

[4] Cronstein BN (1997). The mechanism of action of methotrexate. Rheum Dis Clin North Am.

[5] Brown PM, Pratt AG, Isaacs JD (2016). Mechanism of action of methotrexate in rheumatoid arthritis, and the search for biomarkers. Nat Rev Rheumatol.

[6] van Ede AE, Laan RF, Blom HJ, De Abreu RA, van de Putte LB (1998). Methotrexate in rheumatoid arthritis: an update with focus on mechanisms involved in toxicity. Semin Arthritis Rheum.

[7] Ahmadzadeh A, Zamani N, Hassanian-Moghaddam H, Hadeiy SK, Parhizgar P (2019). Acute versus chronic methotrexate poisoning; a cross-sectional study. BMC Pharmacol Toxicol.

[8] Kivity S, Zafrir Y, Loebstein R, Pauzner R, Mouallem M, Mayan H (2014). Clinical characteristics and risk factors for low dose methotrexate toxicity: a cohort of 28 patients. Autoimmun Rev.

